# Markov chain Monte Carlo based analysis of post-translationally modified VDAC gating kinetics

**DOI:** 10.3389/fphys.2014.00513

**Published:** 2015-01-13

**Authors:** Shivendra G. Tewari, Yifan Zhou, Bradley J. Otto, Ranjan K. Dash, Wai-Meng Kwok, Daniel A. Beard

**Affiliations:** ^1^Department of Molecular and Integrative Physiology, University of MichiganAnn Arbor, MI, USA; ^2^HD Biosciences CorporationShanghai, China; ^3^Department of Anesthesiology, Medical College of WisconsinMilwaukee, WI, USA; ^4^Department of Physiology, Medical College of WisconsinMilwaukee, WI, USA; ^5^Biotechnology and Bioengineering Center, Medical College of WisconsinMilwaukee, WI, USA; ^6^Department of Pharmacology and Toxicology, Medical College of WisconsinMilwaukee, WI, USA

**Keywords:** mitochondria, VDAC, lipid bilayer, electrophysiology, nitrosation, phosphorylation, MCMC

## Abstract

The voltage-dependent anion channel (VDAC) is the main conduit for permeation of solutes (including nucleotides and metabolites) of up to 5 kDa across the mitochondrial outer membrane (MOM). Recent studies suggest that VDAC activity is regulated via post-translational modifications (PTMs). Yet the nature and effect of these modifications is not understood. Herein, single channel currents of wild-type, nitrosated, and phosphorylated VDAC are analyzed using a generalized continuous-time Markov chain Monte Carlo (MCMC) method. This developed method describes three distinct conducting states (open, half-open, and closed) of VDAC activity. Lipid bilayer experiments are also performed to record single VDAC activity under un-phosphorylated and phosphorylated conditions, and are analyzed using the developed stochastic search method. Experimental data show significant alteration in VDAC gating kinetics and conductance as a result of PTMs. The effect of PTMs on VDAC kinetics is captured in the parameters associated with the identified Markov model. Stationary distributions of the Markov model suggest that nitrosation of VDAC not only decreased its conductance but also significantly locked VDAC in a closed state. On the other hand, stationary distributions of the model associated with un-phosphorylated and phosphorylated VDAC suggest a reversal in channel conformation from relatively closed state to an open state. Model analyses of the nitrosated data suggest that faster reaction of nitric oxide with Cys-127 thiol group might be responsible for the biphasic effect of nitric oxide on basal VDAC conductance.

## Introduction

The mitochondrial inner membrane (MIM) and mitochondrial outer membrane (MOM) are the two phospholipid membranes separating the mitochondrial matrix milieu from cytosol. Transport between cytosol and matrix is essential for mitochondrial function. The most abundant protein in the mammalian MOM is a voltage-dependent anion channel (VDAC) (Krimmer et al., 2001; Bayrhuber et al., 2008; Rostovtseva and Bezrukov, 2008). Of the three identified isoforms, VDAC1 is the most highly expressed (De Pinto et al., 2010; Messina et al., 2012; Maldonado et al., 2013). VDAC is the main conduit that connects the cytosol to the mitochondrial inter-membrane space (MIS; the narrow region between MOM and MIM), and allows the passage of carbon substrates (e.g., pyruvate, succinate), phosphates (e.g., ATP, ADP, Pi), and cations (e.g., Ca^2+^, K^+^, H^+^) across the MOM. Because VDAC has been linked to both cell survival and apoptosis, it has been described as the “gatekeeper of mitochondrial function and dysfunction (Kerner et al., 2012).”

By transporting substrates for the tricarboxylic acid cycle and oxidative phosphorylation across the MOM, VDAC plays a significant role in regulating mitochondrial bioenergetics (Hodge and Colombini, 1997; Rostovtseva and Colombini, 1997; Rostovtseva and Bezrukov, 1998; Guzun et al., 2012). In the closed state, the channel is believed to preferentially transport cations and impede the transport of organic anions, including substrates (Tan and Colombini, 2007; Tikunov et al., 2010; Tan, 2012). Thus, VDAC closure may obstruct substrate supply for respiration and prevents exchange of ADP for ATP synthesized by oxidative phosphorylation. As a major gateway in and out of the mitochondria, VDAC mediates an intimate dichotomy between cellular metabolism and cell death (Lemasters and Holmuhamedov, 2006; Shoshan-Barmatz et al., 2008; Tikunov et al., 2010; Mccommis and Baines, 2012). Recent observations indicated that the function of VDAC is regulated by protein-protein interactions as well as by post-translational modifications (PTMs) (Kerner et al., 2012). Moreover, studies show that VDAC undergoes PTM in neurodegenerative diseases such as Huntington's and Alzheimer's diseases (Perluigi et al., 2005; Mello et al., 2007; Ferrer, 2009) and in cardiac ischemia-reperfusion (IR) injury (Das et al., 2012; Kerner et al., 2012). Yet how PTMs of VDAC impacts its function and how that subsequently impacts the mitochondrial function have not been fully characterized.

To study the effects of PTM on VDAC, single channel current recordings of cardiac wild-type (WT) and PTM VDAC reconstituted in planar lipid bilayers are analyzed. Specifically, single VDAC data from two types of PTM are studied: nitrosation of VDAC protein using NO donor PAPA NONOate (PPN) and phosphorylation (phospho-mimetic) of VDAC protein at a serine residue. The nitrosation data is the recently published biphasic effect of PPN on VDAC purified from rat heart (Cheng et al., 2011). The phosphorylated VDAC activity is based on independent recombinant VDAC experiments. The phosphomimetic mutant is constructed after mutating Serine 137 with Glutamic acid. Both these forms of VDAC PTMs have been implicated in cardiac ischemia-reperfusion injury but their effect on VDAC gating kinetics is not well understood.

To better understand the kinetics of VDAC activity obtained from the single channel current recordings under these conditions, mathematical modeling is utilized to develop a kinetic model of the channel activity. In particular, a generalized continuous-time Markov-chain Monte Carlo (MCMC) method, based on Siekmann et al. (2011), is used. MCMC methods extract more information from experimental data compared to maximum-likelihood estimators (MLEs) (Qin et al., 1996, 1997). As compared to MLEs which return single point parameter estimate, MCMC methods return probability distribution of identified model parameter space from which important model information such as, model complexity and appropriateness can be easily obtained.

## Methods

### Purified WT and nitrosated VDAC data

Cheng et al. (2011) studied the effect of exogenous NO on VDAC purified from rat heart mitochondria. They reported a biphasic effect of PPN on VDAC reconstituted in planar lipid bilayers. Peak inhibition was reported at 25 μM PPN (PPN25) which corresponds to a NO concentration of approximately 350 nM. This is within the range of NO that has been shown to be anti-apoptotic. At higher levels of NO, this inhibitory effect was attenuated and eventually enhanced VDAC conductance at 100 μM PPN (PPN100; corresponding to approximately 900 nM of NO). All these experiments were performed at room temperature and command voltages of −10 mV. For detailed methodology for these protocols see Cheng et al. (2011). Here data reported by Cheng et al. (2011) are analyzed to characterize the effect of NO (possibly via nitrosation) on VDAC purified from primary tissue.

### Construction of phosphorylated VDAC

VDAC full length cDNA clone from rat heart with the corresponding GenBank accession number BC072484 was purchased from the Mammalian Gene Collection of Open Biosystems (Huntsville, AL). The coding sequence of VDAC was amplified and cloned by standard PCR methods in the BamHI/Not1 sites of *Escherichia coli* expression vector pET-21a (Novagen, Darmstadt, Germany). Colonies were screened by restriction digestion and the VDAC sequence together with its His-tag at the C-terminus was verified by sequencing. Construction of the VDAC phosphomimetic, where Ser 137 was mutated to Glu, was achieved by overlapping PCR amplification utilizing the QuickChange Site-Directed Mutagenesis Kit (Stratagene, La Jolla, CA). Under physiological conditions, this Ser residue has been shown to be phosphorylated in rat liver VDAC (Distler et al., 2007), and we have also confirmed its basal phosphorylation in rat heart by tandem mass spectrometry using the Thermofinnigan LTQ XL ion-trap mass spectrometer (MS) in the Biotechnology and Bioengineering Center at the Medical College of Wisconsin and analyzed by multistage-fragmentation analysis (Chesnik et al., 2011).

Induction of VDAC expression in BL21(DE3) cells was achieved by incubating the overnight culture for an additional 4 hours after addition of 1 mM isopropyl β-D-10 thiogalactopyranoside. Cells were then pelleted and lysed, and the VDAC containing inclusion bodies were subsequently isolated by centrifugation. The inclusion bodies were dissolved in solubilization buffer and then loaded onto nickel-nitrilotriacetic acid (Ni-NTA) His•Bind Resin (Novagen) columns. Bound proteins were dislodged from the matrix with a Ni-elution buffer. Subsequently, the purified VDAC was refolded by drop-wise dilution in a 1:10 ratio of elution buffer to the refolding buffer. The un-phosphorylated/recombinant VDAC (rVDAC) and phosphomimetic VDAC (S137E) proteins were reconstituted into planar lipid bilayers to measure their respective channel current activities as previously described (Cheng et al., 2011). The term un-phosphorylated is used throughout the text to indicate the recombinant VDAC.

### Reconstitution of VDAC in planar lipid bilayer

Briefly, a mixture of phospholipids in chloroform were mixed and dried under a stream of nitrogen and dissolved in n-decane with a final lipid composition of phosphatidylethanolamine, phosphatidylserine and phosphatidylcholine (Avanti Polar Lipids, Alataster, AL) at a ratio of 5:4:1 (v/v). The lipid bilayers were formed in symmetrical solutions, with both the *cis* and *trans* chambers containing (in mM): 10 HEPES, 500 NaCl, 1 CaCl_2_, 0.2 MgATP, with pH 7.4. The *cis* chamber was held at virtual ground, and the *trans* chamber was held at the command voltages. The VDAC protein was incorporated from the *cis* side. Currents were digitized at 5 kHz and low pass filtered at 1 kHz using a voltage clamp amplifier (Axopatch 200B, Molecular Devices, Sunnyvale, CA) via a digitizer (DigiData 1332, Molecular Devices), and recorded in 1 minute segments at a command voltage of −10 mV. Experiments were performed at room temperature. The pClamp software (version 10, Molecular Devices) was used for data acquisition and analysis is done using a MATLAB based script implementing the developed MCMC algorithm. Although decane-containing membranes have been commonly used in recording the biophysical characteristics of VDAC reconstituted in planar lipid bilayers, it should be noted that the solvent could increase the thickness of the membrane by approximately 1 nm and potentially alter the properties of the channel (Tillman and Cascio, 2003). Despite such limitations, recording VDAC in planar lipid bilayers is a preferred method due to the significant difficulty in measuring VDAC activity in its native environment.

### Continuous-time Markov model of WT and PTM VDAC

**Figure 1:**
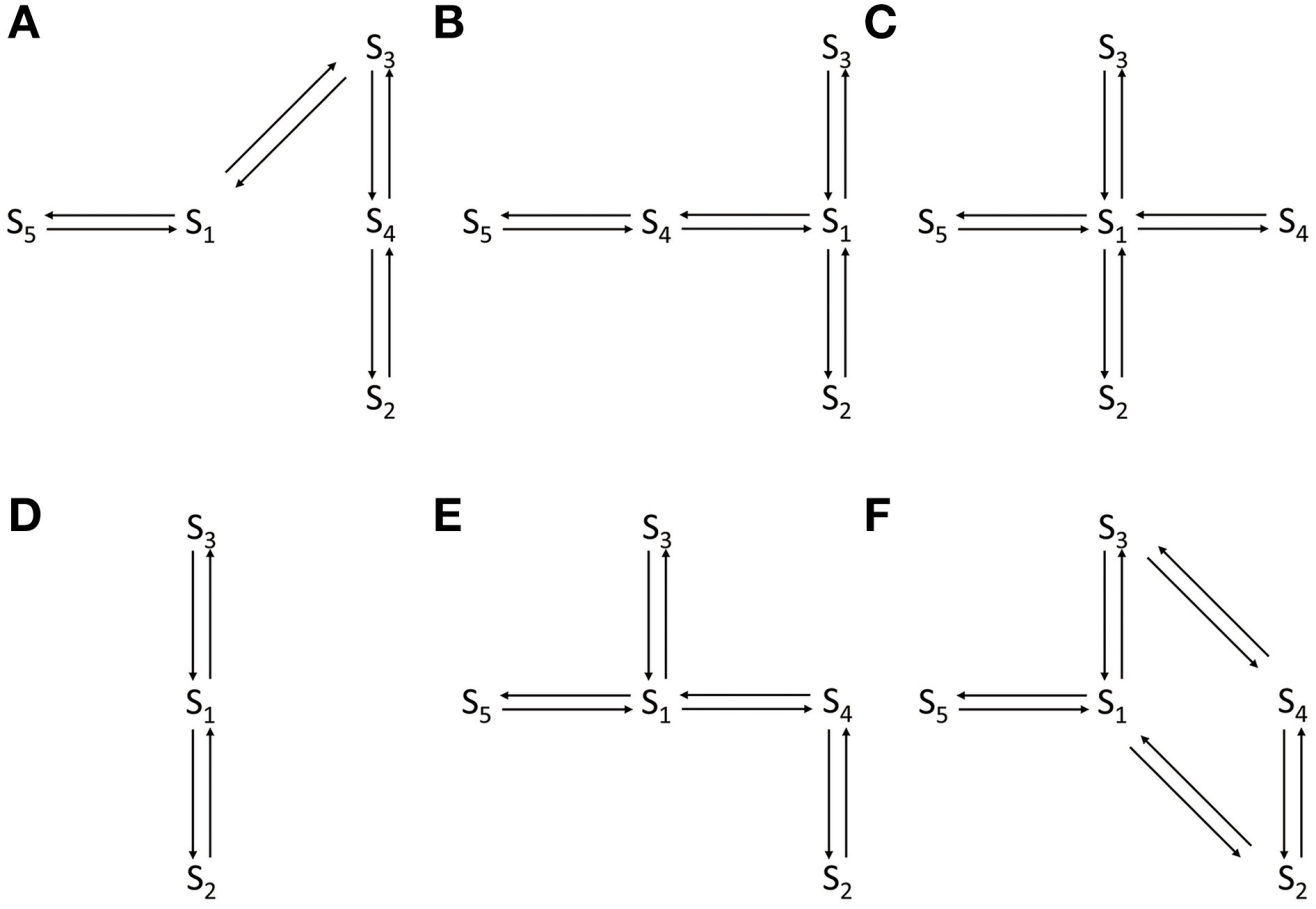
**Different Markov models that were attempted to fit WT and PTMed VDAC data. (A)** The open loop Markov model that could describe the WT and PTM VDAC activity. *S*_1_–*S*_5_ represent distinct VDAC conformations. *S*_3_: Maximally open VDAC conformation; (*S*_5_, *S*_1_, *S*_4_): Sub-conductance state of VDAC (all three have the same conductance level); *S*_2_: Minimally open or closed state of VDAC. **(B–F)** Failed/inappropriate models. In all the Markov models *S*_3_ and *S*_2_ refer to maximally open and minimally open VDAC state, respectively.

The reconstituted VDAC activity recorded from the planar lipid bilayer experiments is sampled with a time-interval of fixed length (τ) and represented as a sequence of discrete-time channel events *I* = (*I*_k_)^N^_k = 1_, where *N* is the number of data-points in the recording and *I*_k_ is the single channel current at time *t*_k_. A typical VDAC current recording has multiple open and closed (or minimally open) states with a dominant sub-state that can be regarded as half-open or a half-closed state. Thresholding of VDAC current is employed to identify channel openings and closures which are classified into three distinct conductance states: open (*O*), sub-state (*O*_S_), or closed (*C*) event:

(1) $$E_{\text{k}}=E(t_{\text{k}})=\{\begin{array}{l} O,\text{ if }\left|I_{\text{k}}\right|>\left|I_{\text{O}}\right|,\\ C,\text{ if }\left|I_{\text{k}}\right|<\left|I_{\text{C}}\right|,\\ O_{\text{S}},\text{ otherwise}, \end{array}$$

where |*I*_O_| > |*I*_C_|. The thresholds *I*_O_ and *I*_C_ are set at fixed values to identify distinct VDAC conductance states. These threshold values are different for WT and PTMed VDAC activity, however were chosen to be same within a given data type (i.e., WT, S137E, etc.). The threshold values used are tabulated in Table 1. The event sequence (*E*_k_)^N^_k = 1_ obtained in Equation 1 can be represented by a Markov model. A Markov model is a symmetric directed graph. The set of vertices contain distinct Markov states {*S*_1_, *S*_2_, … *S*_n_} and the set of edges contain the non-negative constants *q*_ij_ (i,j: 1, 2, …, n) that govern the transition rate from state *S*_i_ to state *S*_j_. Figure 1 shows different Markov models.

**Table 1:** **Threshold values and parameters estimated for WT and PTM VDAC activity**.

	**WT (*n* = 4)**	**PPN 25 μM^a^ (*n* = 5)**	**PPN 100 μM^a^ (*n* = 5)**	**rVDAC (*n* = 3)**	**S137E (*n* = 3)**
**THRESHOLD VALUES (pA)**					
*I*_C_	−20	−9	−38	−21.5	−20
*I*_O_	−15	−6.7	−34	−14.5	−15
**RATE CONSTANTS (s^−1^)^b^**					
*q*_13_	141.6 ± 6.4	230 ± 1.8	120 ± 1.3	130.3 ± 14.8	135.3 ± 2.59
*q*_31_	9.79 ± 4.8	3 ± 1.3	0.66 ± 0.27	47.5 ± 7.8	1.26 ± 0.49
*q*_15_	177.3 ± 9.7	95 ± 1	139 ± 1.3	118.6 ± 14.8	152.2 ± 9.13
*q*_51_	148.1 ± 5.5	220 ± 1.1	118 ± 1	161.2 ± 8	137.4 ± 20.17
*q*_34_	253.3 ± 11.0	110 ± 1.1	157 ± 0.85	237.2 ± 8.1	169.9 ± 2.5
*q*_43_	0.24 ± 0.12	0.12 ± 0.21	0.094 ± 0.02	0.36 ± 0.33	0.61 ± 0.2
*q*_24_	332.6 ± 9.5	0.46 ± 0.13	165 ± 7	296.1 ± 13	143.8 ± 19.8
*q*_42_	0.4 ± 0.07	0.048 ± 0.014	0.075 ± 0.016	3.6 ± 0.8	0.83 ± 0.29

a *These parameters are estimated by simultaneous fitting of all the dataset within each data-type*.

b *These rate constant values are the mean of the representative parameters, such as shown in **Figure 4B**, obtained after fitting the Markov model against the WT and PTM VDAC data*.

In general, a given channel event can be represented by more than one Markov state. For example, *S*_5_, *S*_1_, *S*_4_ represent the same event *O*_S_ for the Markov model shown in Figure 1A. Therefore different Markov sequences (*M*_k_) can yield same event sequence (*E*_k_). To circumvent this problem and identify the best Markov model, Siekmann et al. (2011) construct a probability distribution *P*((*M*_k_)|(*E*_k_), *Q*), where *Q* is known as the infinitesimal generator of a continuous-time Markov model. It is a matrix consisting of entries given by *q*_ij_ (*i* ≠ *j*) and zero when two states are not connected. The diagonal entries of the matrix *Q* are given by:

(2) $$q_{\text{ii}}=-\sum_{i\;\neq \;j}q_{\text{ij}},i\;=\;1,\ldots ,n.$$

Let *p*_0_ be vector containing the initial probability distributions for the set of Markov states. Then the time evolution of the Markov state probabilities, *p*(*t*), can be calculated by the following differential equation:

(3) $$\frac{dp(t)}{dt}=Qp(t)\Rightarrow p(t)=\text{exp}\left(Qt\right)p_{\text{0}},$$

where exp denotes matrix exponential which is computed using the Pade approximation (Arioli et al., 1996). Using Equation 3 the transition probability from state *S*_i_ to *S*_j_ in a given sampling interval (τ) is computed:

(4) $$A_{\tau }=\text{exp}\left(Q\tau \right)=a_{\text{ij}},i,j:1,\ldots ,n.$$

To account for different conducting states of VDAC activity the Siekmann et al. (Siekmann et al., 2011) method is modified to introduce three projection vectors θ_O_, θ_OS_, θ _C_ which correspond to the three possible events i.e., *O, O*_S_, *C*. The purpose of these projection vectors is to restrict transition from state *S*_i_(*t*_k−1_) to *S*_j_(*t*_k_) if *S*_j_(*t*_k_) ∉ *E*_k_. This is done by modifying the transition probability matrix *A*_τ_ according to an event at a given time *t*_k_, such that:

(5) $${A_{\tau }|}_{t=t_{\text{k}}}=A_{\tau }\theta_{\text{O}}\text{ if }E\left(t=t_{\text{k}}\right)=O.$$

Such projection matrices are useful in developing Markov models of channels with multiple sub-conducting states such as, VDAC. Here, the Metropolis–Hastings (MH) algorithm is used for sampling different Markov models (Brooks, 1998; Siekmann et al., 2011). MH algorithm is basically a two-step process:

- Random walk MH updating is used to generate a new model candidate $\tilde{Q}$ with realizations of the form $\tilde{q}$_ij_ = *q*_ij_ + *u*_ij_, *u*_ij_ ∈ *U*{−δ, δ}. Where, *U*{−δ, δ} is a continuous uniform distribution on the interval [−δ, δ]. It is also required that the new model satisfies the condition of detailed balance: π_i_*q*_ij_ = *q*_ji_ π_j_. Here, π is the stationary distribution to which Markov state probabilities *p*(*t*) tend as *t* → ∞. The detailed balance condition is automatically satisfied if $\tilde{Q}$ is acyclic (Siekmann et al., 2011).
- The candidate model $\tilde{Q}$ is accepted with probability.
  (6) $$\alpha =\min\left\{1,\frac{P\left(\tilde{Q}\right)P\left(M|E,\tilde{Q}\right)}{P\left(Q\right)P\left(M|E,Q\right)}\right\}.$$
  Here, $P\left(Q\right)=\text{exp}\left(\frac{\sum_{\forall i}q_{\text{ii}}}{\lambda }\right)$ is the prior distribution that expresses a belief or information about the parameters beforehand (Rosales et al., 2004). For this choice of prior distribution, the parameter values are assigned a low probability when they become much larger than λ. The MCMC algorithm is relatively insensitive to this parameter therefore its value was fixed at 30 ms^−1^ for all the VDAC dataset—unless very small values are used (Siekmann et al., 2011). It is clear from Equation 6 that a new Markov model is accepted if its likelihood for the event sequence is greater than the previous trial model.

Independent runs of the algorithm tested different competing Markov models (shown in Figure 1). The best model is chosen as the one that explains all data from all of the experiments on the four different form of VDAC analyzed and based on the uncertainty in the associated parameter distributions. The model that is selected (shown in Figure 1A) invokes a total of eight parameters which are found to be different for WT, nitrosated, un-phosphorylated and phosphorylated VDAC recordings.

### Open-time, half-open-time, and close-time distributions

To validate the robustness of the Markov model, open-time, half-open-time, and close-time distribution of the VDAC activity are computed and are compared with the open-time, half-open-time, and close-time distributions derived from the experimental data. These probability density functions govern the time for which the channel remains in open state, half-open state or closed state. Colquhoun et al. (Colquhoun and Hawkes, 1981, 1982) derived expressions for these probability distributions making use of the infinitesimal generator *Q*. In general, the probability density function of open times can be written as:

(7) $$f(t)=\Phi_{0}\text{exp}\left(Q_{\text{AA}}t\right)\left(-Q_{\text{AA}}\right)u_{\text{A}};\Phi_{0}=\pi_{\text{B}}Q_{\text{BA}}/\pi_{\text{B}}Q_{\text{BA}}u_{\text{A}}.$$

Here, “A” is the subset of Markov states representing only the VDAC open states, *Q*_AA_ = {*q*_ij_ : *i, j* ∈ A} and *u*_A_ is a column vector with *n*_A_ entries which are all equal to one. Φ is the initial (row) vector that defines the relative probability of an opening starting in a given Markov open state. π_B_ is the stationary distribution of the Markov states which do not represent the VDAC open state and *Q*_BA_ = {*q*_ij_ : *i* ∈ B, *j* ∈ A}. In the event when there is just one open state (such as shown in Figure 1A) then both Φ_0_ and *u*_A_ are unity and can be omitted, and the distribution reduces to a simple exponential distribution. For a more comprehensive detail of this approach the reader is referred to Colquhoun and Hawkes (1981, 1982).

## Results

We applied the generalized continuous-time MCMC method, using the Markov model shown in Figure 1A, for the electrophysiological recordings of WT, nitrosated, un-phosphorylated and phosphorylated VDAC activity. The Markov model is used to compute convergence plots of the parameters and their posterior probability distributions which tell us about the goodness of the model and the parameter estimates. Other competing Markov models, listed in Figures 1B–F, were ruled out because they could not fit the PPN25 VDAC current data. The reasons for this are reviewed in the section Discussion.

### Model simulations of purified WT and nitrosated VDAC

**Figure 2:**
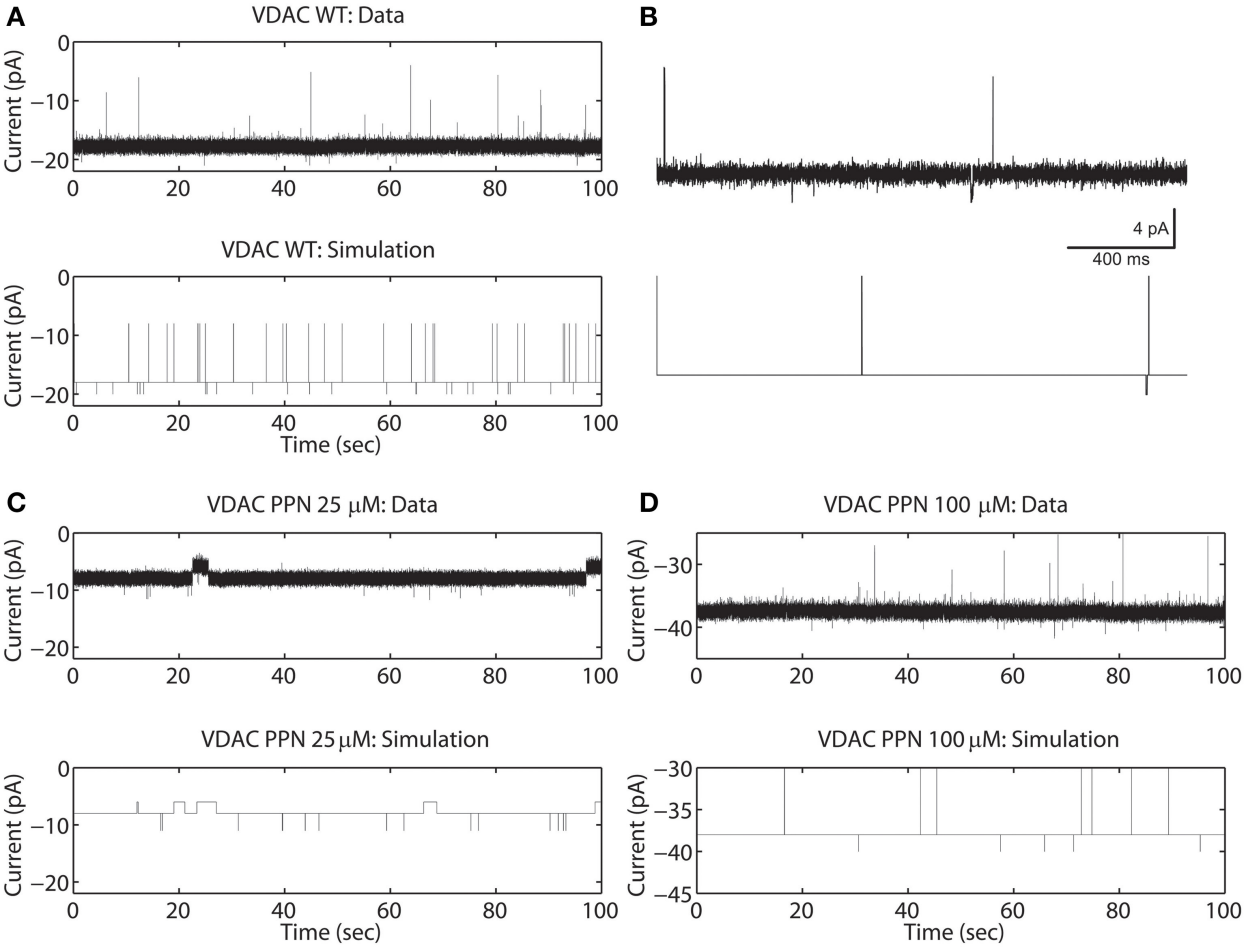
**Representative data and simulations for the WT (A), PPN 25 μM **(C)**, and PPN 100 μM **(D)** VDAC activity in response to a holding potential of −10 mV**. Panel **(B)** 2 second long trace of data and model simulated data of WT VDAC activity. Downward deflections indicate open channel events and upward deflections indicate channel closure events. Nitrosation of VDAC by PPN 25 μM and 100 μM evoke robust changes in the VDAC conductance and gating kinetics. The model successfully reproduces the burst seen in PPN 25 μM data. The model also successfully reproduces open and closed channel events as identified by the threshold. In experimental data open and closing events appear on a background measurement noise of ±0.5 pA (standard deviation). Measurement noise is not present in model simulations.

In the current recordings analyzed here, the VDAC activity has at least three conducting states which are identified using the thresholds (*I*_O_ and *I*_C_). For example, the WT VDAC data shown in Figures 2A,B has three different conducting events which for the Markov model shown in Figure 1A correspond to: open state S_3_ if |*I*(*t*_k_)| ≥ (|*I*_O_| = 20) or closed state S_2_ if |*I*(*t*_k_)| ≤ (|*I*_C_| = 15) or else half-open state(s) *S*_1_, *S*_4_, *S*_5_. Similarly, distinct conducting events for nitrosated VDAC data are also identified using the threshold values listed in Table 1. It is apparent from the purified WT VDAC data (see Figure 2A) that the channel openings and closings are variable in amplitude but model simulations have fixed discrete conductance (see Figure 2A). The simulations assume three distinct conductance states (closed, half-open or open) to match VDAC opening or closing identified using the threshold values.

It is apparent from Figure 2A that the model is successfully able to capture the relative frequency of transitions of WT VDAC to the open and closed state which is apparent from the short 2-second long trace of WT VDAC data and model simulation (see Figure 2B). Nitrosation of VDAC by PPN induces robust changes in its conductance and kinetics which are also successfully captured by the Markov model (see Figures 2C,D). The marked reduction and increase in basal VDAC conductance are captured by changing model VDAC conductance in an ad-hoc manner as described above. The model simulations are able to qualitatively reproduce purified WT and nitrosated VDAC kinetics. The burst-like behavior with PPN 25 μM (see Figure 2C) and WT-like behavior with PPN 100 μM (see Figure 2D) are both successfully emulated by the Markov model. The mean values of the estimated parameters for the two data types are listed in Table 1 and shown in **Figure 7A**.

### Model simulations of un-phosphorylated and phosphorylated VDAC

**Figure 3:**
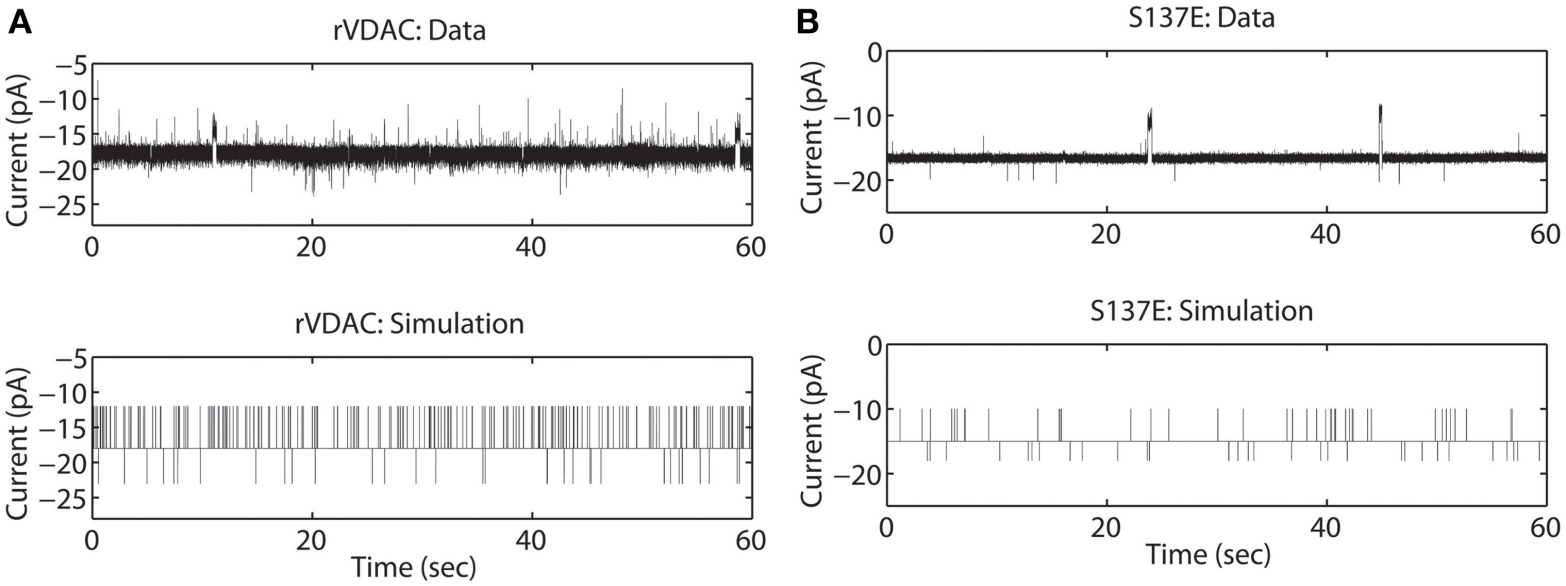
**Representative data and simulations for the un-phosphorylated (rVDAC; **A**) and phosphorylated (S137E; **B**) VDAC activity**. The un-phosphorylated VDAC data exhibits more closures (as compared to the purified or WT VDAC) and phosphorylated VDAC data exhibits more openings (less closures) which are accurately replicated by the model simulations. In experimental data open and closing events appear on a background measurement noise of ±0.5 pA (standard deviation). Measurement noise is not present in model simulations.

The rVDAC activity behaves differently than the purified or WT VDAC activity. Compare the data shown in Figures 2A, 3A. In particular, the rVDAC tends to close more rapidly than the purified WT VDAC. To ensure that the model parameter estimates of the rVDAC data are minimally affected by background noise, the thresholds for closed and open states were slightly increased (compared to purified WT). While determining model parameter estimates of the S137E data, the threshold values were not changed as the background noise level is low (see data shown in Figure 3B). The model simulations of un-phosphorylated (see Figure 3A) and phosphorylated data (see Figure 3B) match the experimental data reproducing the marked increase and decrease in VDAC activity. The rVDAC data and simulation show a high probability of closing while the S137E data and simulation show a low probability of closing. Again, VDAC conductance levels (openings and closings) during model simulations are assigned in a manner similar to the purified WT and nitrosated data. The mean values of the estimated parameters for the two data types are listed in Table 1 and shown in **Figure 7B**.

### Parameter convergence plots & posterior distributions

**Figure 4:**
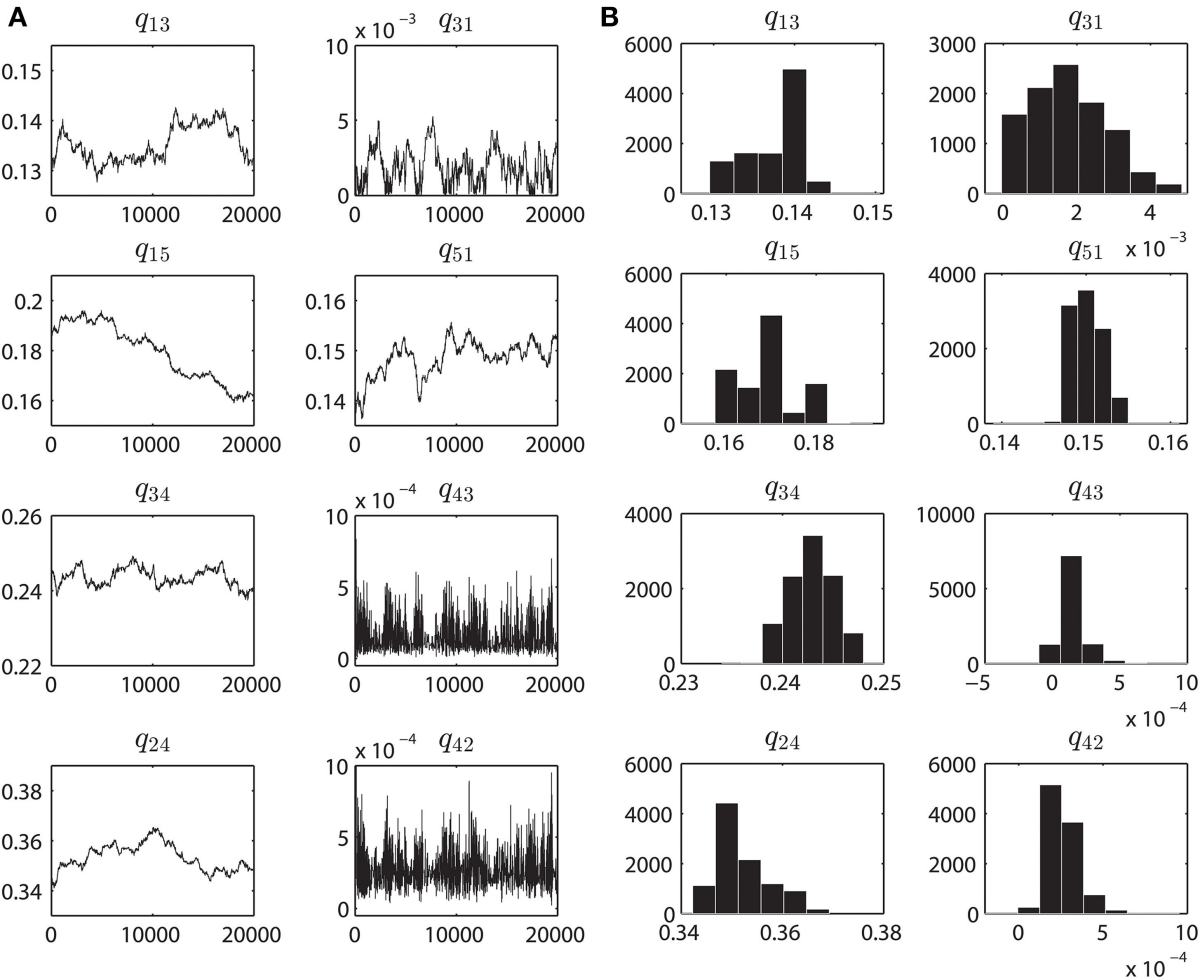
**Parameter convergence plots and Posterior distributions. (A)** MCMC results when inferring the parameters from the purified WT VDAC activity consisting of 500,000 data points. Eight panels correspond to the eight rate constants, as seen in Figure 1A, governing the VDAC conformation changes. For all the data-sets the MCMC method was ran for 20,000 iterations. The initial estimates of the parameters are result of multiple runs of MCMC method (with different values of δ) for 10K iterations to determine the neighborhood of best parameters. Ordinate unit is milliseconds. **(B)** Histograms computed from the MCMC results shown in **A**. Prior to calculating the histograms, first 10000 iterations were discarded as the burn-in period. These histograms provide an estimate of the accuracy of identified parameters. One single hump evident from the sub-panels signifies the optimal parameter value which was chosen over other parameters based on Bayesian inference. Abscissa unit is milliseconds.

The parameter convergence plots are shown in Figure 4A which are obtained from fitting the WT VDAC data, with the Markov model shown in Figure 1A, using the MCMC approach described earlier. The initial estimate of the parameters was obtained by multiple runs of the MCMC method for 10,000 iterations with variable values of δ ∈ [0.0005, 0.05]. This approach helped in obtaining a reasonable initial estimate of model parameters. Posterior probability distributions from the convergence plots are also computed. While computing the posterior distributions of the parameters, first 10,000 iterations are discarded as the burn-in period. Posterior distributions of the parameters provide valuable information regarding the Markov model e.g., model complexity and goodness of the model fits. Figure 4B shows the posterior distributions computed from the convergence plots obtained by fitting purified WT VDAC activity. All of the rate constant histograms have one pronounced peak which represents the best model parameter values. WT VDAC is known to reside predominantly in an open sub-state (*S*_4_ in our model), therefore transitions to a higher conductance state (*S*_3_) or a lower conductance state (*S*_2_) seldom happen. The model captures this aspect of WT VDAC.

### Dwell-time distributions & stationary probabilities

**Figure 5:**
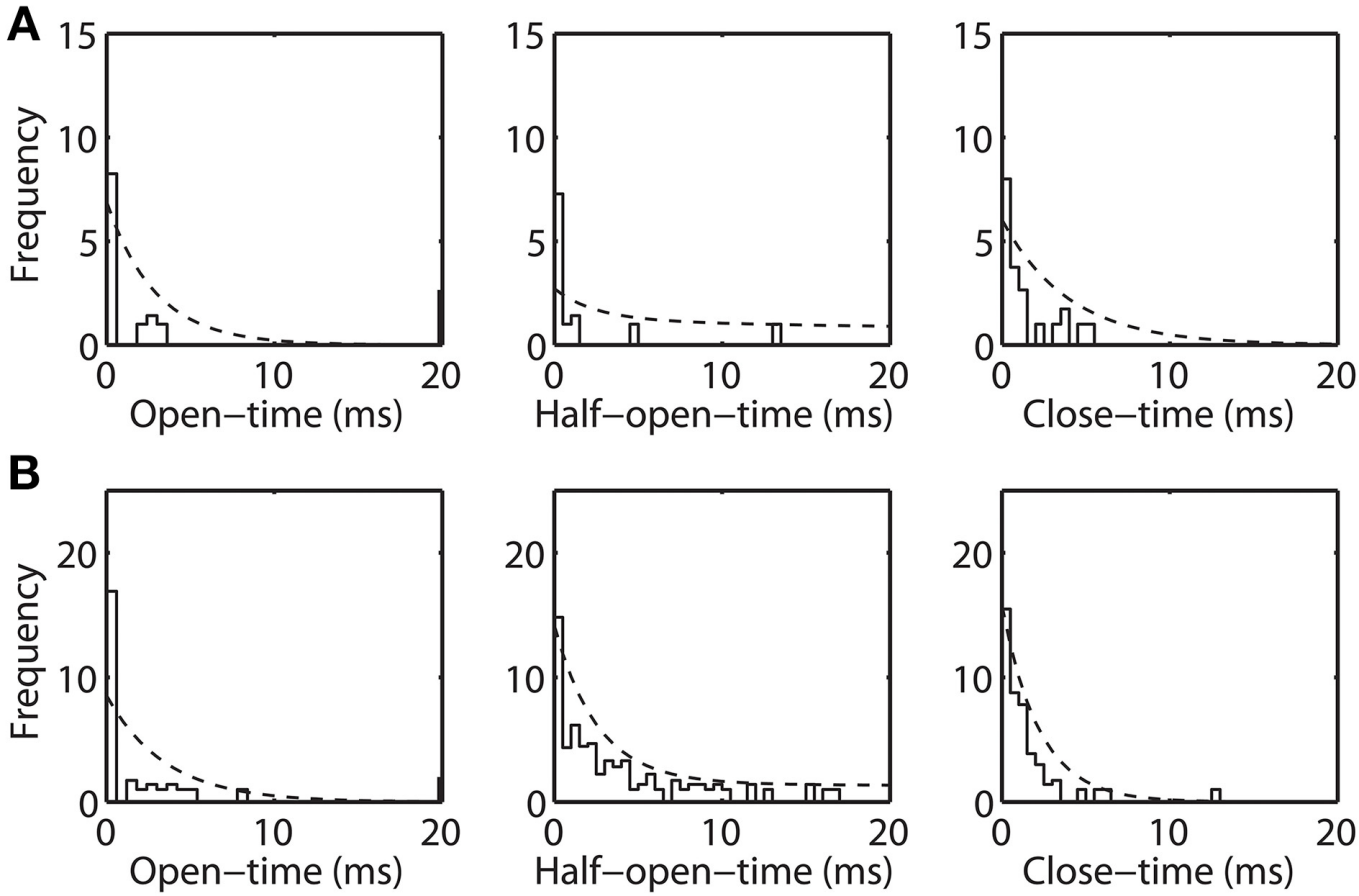
**Open-time, half-open-time, and close-time distributions inferred from the Markov model and the experimental data**. Frequency is reported in square-root scale as previously suggested (Colquhoun et al., 1996). Histogram bin-width is 0.5 msec. Dashed line is the theoretical distribution (η · *f* (*t*)), where η is the number of open, half-open and closed events and *f* (*t*) is as shown in Equation 7. Panels **(A)** and **(B)** shows the experimental and theoretical distribution for the purified WT and recombinant VDAC highlighting their kinetic differences.

The open-time, half-open-time, and close-time distributions are computed from the experimental data (shown in Figure 5). These dwell-time distributions are represented by (square-root transformed) frequency, similar to (Colquhoun et al., 1996), of observing an event of given duration. These dwell-time distributions are compared with the theoretical distribution (η · *f* (*t*)) inferred from the Markov model. Here, η is an adjustable parameter accounting for total number of a given event, since very brief events may escape detection due to limitations in the experimental data time-resolution (Lauger, 1988). Dwell-time distributions calculated for purified (Figure 5A) and recombinant VDAC (Figure 5B) confirm the kinetic differences suggested from their single channel recordings. Indeed, rVDAC closes more frequently and has longer lived event durations. Notice the differences in the ordinate limits in Figures 5A,B. The theoretical dwell-time distribution (dashed line) matches well with the dwell-time distributions inferred from the experimental data (see Figures 5A,B).

**Figure 6:**
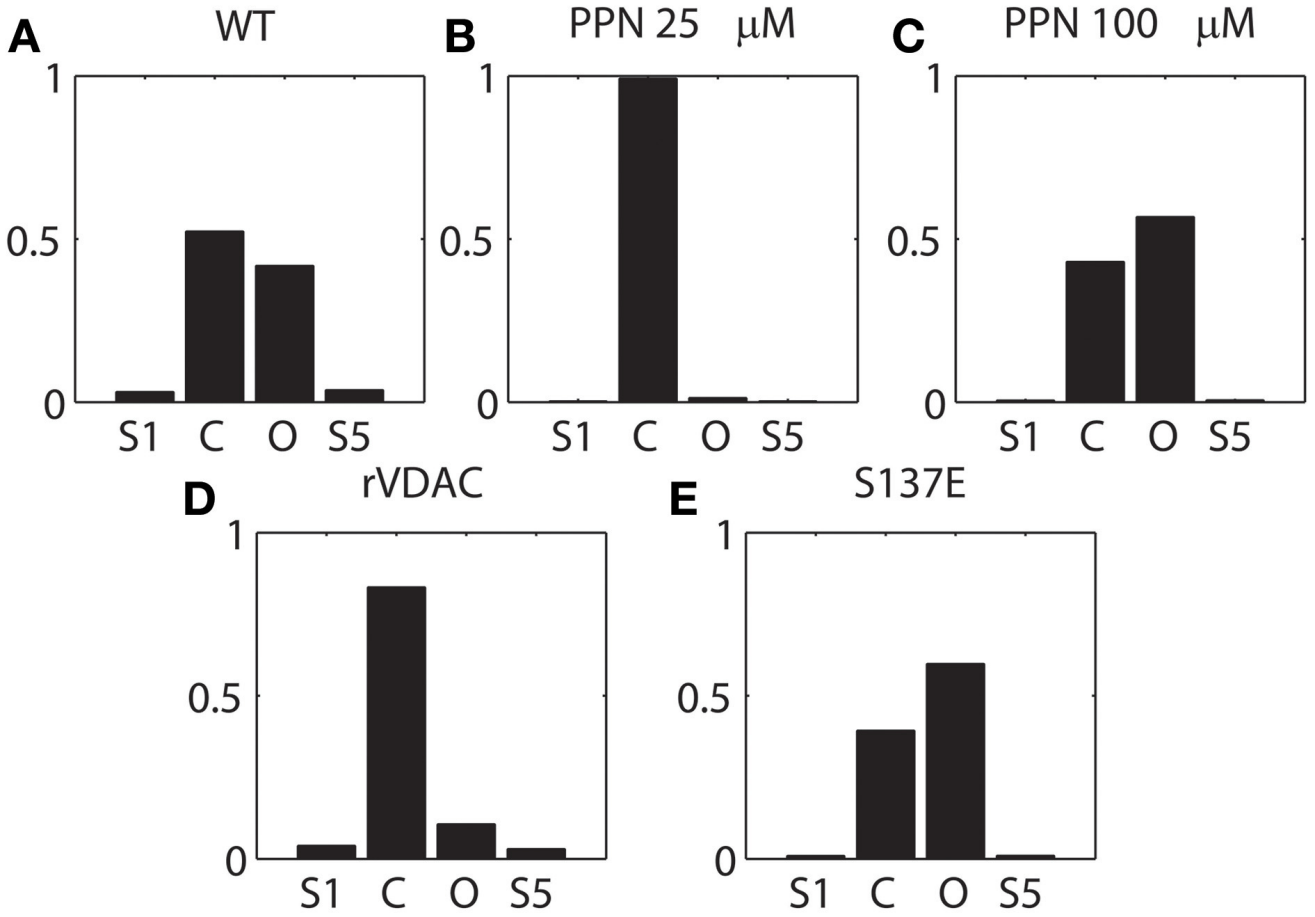
**Stationary distribution of the VDAC states shown in Figure 1A. (A–C)** describes the distribution of the WT and nitrosated VDAC, and **(D, E)** describe the un-phosphorylated (rVDAC) and phosphorylated VDAC (S137E). *S*_4_ was omitted from computations because it seemed to be the dominant dwell state which did not change significantly with different conditions. Y-axis: probability (unitless); X-axis: C and O correspond to *S*_2_ and *S*_3_, respectively.

It is apparent from Figure 2 that PPN 25 μM induced significant changes to the VDAC gating kinetics. In order to quantify these changes we calculated the stationary distributions of the Markov model following each condition WT and PTMs. The stationary distribution suggested that the most dominant Markov state of VDAC is *S*_4_ irrespective of the type of experimental data (WT or PTM). Therefore, it is excluded from the distributions shown in Figure 6 to understand relative dwell-time changes among states: (*S*_1_, *S*_2_, *S*_3_, *S*_5_). Stationary distributions, shown in Figure 6, are the solution of π*Q* = 0, *Q* is the infinitesimal generator of Markov chains describing WT and PTM VDAC activities. WT VDAC (Figure 6A) and PPN100 VDAC (Figure 6C) distributions suggest minimal changes in the dwell-times of the four states. On the other hand, there is a contrasting change in the dwell-time distribution for the PPN25 VDAC gating kinetics (see Figure 6B). The dwell-time distribution suggests that VDAC stays ~99% of the time in its closed state (*S*_2_) as compared to other states (*S*_1_, *S*_3_, *S*_5_). Un-phosphorylated and phosphorylated VDAC also share significant differences in their stationary distributions. The S137E data based stationary distributions (see Figure 6E) indicate that there is an appreciable increase in the number of open events, evident from the increase in dwell-time probability of the open state as compared to the control that remains predominantly closed (see Figure 6D). Apparently, nitrosation and phosphorylation of VDAC induce significant changes in its gating kinetics, both of which may have profound effects on mitochondrial (dys)function.

### Estimated parameters

**Figure 7:**
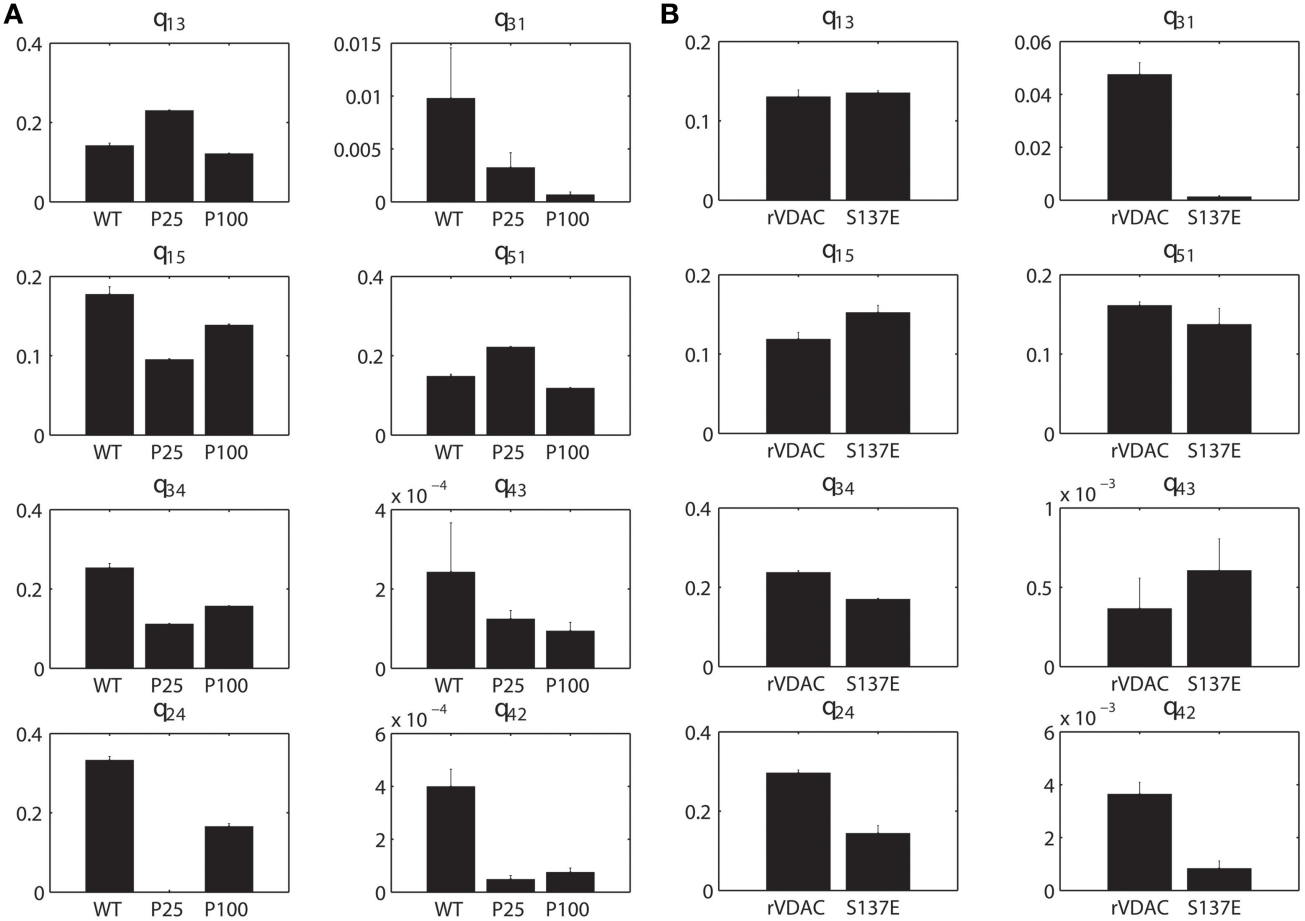
**Estimated parameters for the purified and recombinant VDAC. (A)** Purified Wild-type (WT; *n* = 4), PPN 25 μM (PPN25; *n* = 5) and PPN 100 μM (PPN100; *n* = 5), and **(B)** recombinant WT (rVDAC; *n* = 3) and phosphorylated (S137E; *n* = 4) VDAC. Parameter estimates are calculated from the posterior distributions for each individual data set. A representative distribution for the purified WT VDAC data is shown in Figure 4B. The mean values of estimated parameters are listed in Table 1. Ordinate unit is milliseconds.

In Figures 7A,B, the mean (with standard error) taken over the parameters estimated from each individual electrophysiological data for a WT, nitrosated, un-phosphorylated or phosphorylated VDAC activity is shown. As purified and recombinant VDAC activities are found to be noticeably different (see Figures 5A,B), purified and recombinant data are shown separately for comparison purposes (Figures 7A,B, respectively). Figure 7A shows purified VDAC associated parameters: WT (Wild-type; *n* = 4), P25 (PPN 25 μM; *n* = 5) and P100 (PPN 100 μM; *n* = 5), and Figure 7B shows recombinant VDAC associated parameters: rVDAC (un-phosphorylated; *n* = 3) and S137E (phosphorylated VDAC; *n* = 3). Overall, the estimated parameters are relatively tight. Most significant changes in the estimated parameters of nitrosated VDAC occurred for the PPN 25 μM data. In particular, *q*_13_ and *q*_51_ have significantly increased, and *q*_31_, *q*_24_ and *q*_42_ have significantly decreased. As a result of these changes the PPN25 VDAC gating kinetics are much faster, as compared to purified WT and PPN 100 μM VDAC data. The phosphorylated VDAC (S137E) closes much less as compared to the recombinant VDAC. This behavior is captured by the significant differences in the *q*_31_, *q*_34_, *q*_24_, and *q*_42_ parameters of both the data types (see Figure 7B).

## Discussion

Conduction kinetics of single VDAC was analyzed to identify a Markov model for the channel. The identified model was used to probe the effects of (possible) nitrosation on purified WT VDAC activity via NO donor PPN. The model was also used to study the effect of phosphorylation at a serine residue (phosphor-mimetic) on recombinant VDAC activity. To analyze current recordings from single channels, the statistical method of Siekmann et al. (2011) was modified to account for multiple conducting states of the VDAC. The statistical method makes use of Bayesian inference and stochastic search methods known as MCMC which return more information than maximum likelihood estimators (Qin et al., 1996, 1997). Parameter profiles associated with the best Markov model (see Figure 1A) for WT and PTM VDAC electrophysiology data are listed in Table 1. Also, in order to demonstrate the robustness of the identified model, we also computed open-time, close-time, and half-open-time distributions and compared with the time-distributions derived from experimental data.

Several competing Markov models, shown in Figures 1B–F, were discarded based on their inability to reproduce burst-like behavior of PPN25 VDAC activity (see Figure 2C) and predict the experimentally derived dwell-time distributions (see Figure 5). Among the analyzed PPN25 data not all possessed the burst-like behavior, out of the five data set two possessed bursts and three didn't exhibit burst. It was a characteristic which was not exhibited by any of the attempted Markov model shown in Figures 1B–F. However, the chosen model could exhibit the randomness of burst-like PPN25 VDAC activity. The randomness in producing burst-like activity by the Markov model shown in Figure 1A comes due to the necessary path of traversing from half-open state (*S*_1_) to closed state (*S*_2_) via open state (*S*_3_) and half-open state (*S*_4_).

Comparison of parameter profiles for rVDAC and S137E data suggests that phosphomimetic mutant has lower rate of transition to the closed states (*S*_2_) and higher rate of transition to the open state (*S*_3_). Although not obvious from the raw experimental data, the phosphomutant has a higher probability of being in the open state as compared to the closed state. This phenomenon is revealed by computing the stationary distributions of the Markov model associated with rVDAC and S137E data (see Figures 6D,E). A recent study suggests that phosphorylation of VDAC increases the inhibitory effect of tubulin (Sheldon et al., 2011). The tubulin blocked state of VDAC not only has a decreased conductance but also changes ion-selectivity. In fact, no ATP is transported under tubulin blockage (Gurnev et al., 2011). Thus it is possible that phosphorylation of VDAC can have opposite effects depending upon the absence/presence of endogenous modulators (e.g., tubulin) of VDAC.

The parameter profiles of the purified WT and nitrosated VDAC suggest differences in the kinetics of the VDAC nitrosated using PPN 25 μM and PPN 100 μM. The kinetics of PPN100 VDAC is similar to WT VDAC, while the conductance of the WT VDAC is about half of PPN100 VDAC. The kinetics of PPN25 VDAC is different than WT or PPN100 VDAC. PPN 25 μM not only reduces the conductance of VDAC but it also significantly increases the dwell-time of VDAC in its closed state (~99%; see Figure 6B) and VDAC has only two cysteine residues: one facing the lipid interface (Cys-127) and other facing the internal pore (Cys-232) (Geula et al., 2012). It is possible that covalent incorporations of NO into the two thiol group occurs at different rates. Recent studies also suggest that Cys-127 decreases VDAC conductance (Aram et al., 2010; Geula et al., 2012) and Cys-232 promotes VDAC dimerization (Aram et al., 2010; Geula et al., 2012). Therefore, it is plausible to say that Cys-127 is nitrosated at a low NO as compared to Cys-232. Further experiments (such as, biotin-switch) will need to be performed to confirm if NO is indeed incorporated differentially at the two thiol groups.

While the MCMC method reveals information on the gating kinetics of WT VDAC and VDAC that underwent PTMs, it does not reveal how the VDAC conductance is modified as a result of different PTMs. This is an important aspect of VDAC activity as changes in VDAC conductance not only signify a change in conductance but also changes in ion selectivity. Previously, alternative methods like molecular dynamic simulations have been applied on VDAC (Krammer et al., 2011, 2013; Rui et al., 2011) to study its ion selectivity and conductivity but these methods neither provide information about the gating kinetics nor they are computationally efficient. On the other hand, such details can be added implicitly to the proposed Markov model that will explain the ion selectivity and conductance changes due to PTMs.

Although the proposed model is based on experiments performed at −10 mV, the VDAC current-voltage relationship remains linear at voltages between −30 and +30 mV (Colombini, 2004; Pavlov et al., 2005; Cheng et al., 2011). Therefore, it is expected that the open probability of the channel remains constant and that the kinetics is not strongly sensitive to voltage within this range. The model is not expected to be valid at voltages higher than +30 mV and lower than −30 mV where a change in VDAC ion-selectivity has been observed (Colombini, 1980; Peng et al., 1992).

It is thought that solutes and metabolites across MOM via VDAC can freely enter the MIS so that there is no ionic gradient across the membrane. This rationale has been supported by two observations: VDAC remains open most of the time at low voltages (~10 mV) and its big pore size (2.5–3 nm). VDAC undergoes transitions to lower conducting state (change in ion selectivity) when voltage is increased beyond ±30 mV but the potential mechanisms of MOM potential generation are not known. However, several mechanisms have been proposed for generation of an electrostatic potential maintenance of ionic gradients across the MOM (Colombini, 2012; Lemeshko, 2014). More recently, the role of cytoskeleton protein tubulin on phosphorylated VDAC suggested a physiological role for VDAC in modulating mitochondrial function (Rostovtseva et al., 2008). Here, it is shown that phosphorylation and nitrosation of VDAC invokes significant changes to the biophysical properties of single VDAC activity.

### Conflict of interest statement

The authors declare that the research was conducted in the absence of any commercial or financial relationships that could be construed as a potential conflict of interest.
